# Maternal obesity-induced endoplasmic reticulum stress causes metabolic alterations and abnormal hypothalamic development in the offspring

**DOI:** 10.1371/journal.pbio.3000296

**Published:** 2020-03-12

**Authors:** Soyoung Park, Alice Jang, Sebastien G. Bouret

**Affiliations:** 1 The Saban Research Institute, Developmental Neuroscience Program, Children’s Hospital Los Angeles, Los Angeles, California, United States of America; 2 Inserm, Laboratory of Development and Plasticity of the Neuroendocrine Brain, Jean-Pierre Aubert Research Centre, Lille, France; 3 University of Lille, FHU 1,000 Days for Health, Lille, France; Columbia University, UNITED STATES

## Abstract

The steady increase in the prevalence of obesity and associated type II diabetes mellitus is a major health concern, particularly among children. Maternal obesity represents a risk factor that contributes to metabolic perturbations in the offspring. Endoplasmic reticulum (ER) stress has emerged as a critical mechanism involved in leptin resistance and type 2 diabetes in adult individuals. Here, we used a mouse model of maternal obesity to investigate the importance of early life ER stress in the nutritional programming of this metabolic disease. Offspring of obese dams developed glucose intolerance and displayed increased body weight, adiposity, and food intake. Moreover, maternal obesity disrupted the development of melanocortin circuits associated with neonatal hyperleptinemia and leptin resistance. ER stress-related genes were up-regulated in the hypothalamus of neonates born to obese mothers. Neonatal treatment with the ER stress-relieving drug tauroursodeoxycholic acid improved metabolic and neurodevelopmental deficits and reversed leptin resistance in the offspring of obese dams.

## Abstract 2

Maternal obesity predisposes offspring to develop obesity and type 2 diabetes in later life, but the mechanisms are unclear. This study shows that the metabolic and hypothalamic defects associated with maternal obesity involve high levels of endoplasmic reticulum stress during critical periods of growth and development.

## Introduction

A major shift in our nutritional environment has greatly contributed to the recent obesity epidemic. There is growing evidence that adverse fetal and early postnatal environments increase the risk of developing obesity. In particular, accumulative evidence from both human and animal studies demonstrated that exposure to maternal obesity predisposes offspring to obesity and other metabolic dysfunctions later in life [1,2,3]. The hypothalamus is involved in the control of food intake and energy expenditure. Its development is greatly influenced by the maternal and postnatal nutritional environment [1,2,4,5,6,7]. Primary importance has been given to the arcuate nucleus of the hypothalamus (ARH) because it contains 3 main neuronal populations that play a major role in energy homeostasis: anorexigenic pro-opiomelanocortin (POMC)-expressing neurons, orexigenic neuropeptide Y (NPY), and agouti-related peptide (AgRP)-expressing neurons. The adipocyte-derived hormone leptin directly targets these neuronal populations to cause weight loss effects by stimulating POMC neurons while inhibiting AgRP/NPY neurons. Leptin also promotes the development of POMC and AgRP/NPY axonal projections during early postnatal life [8]. Prior studies have shown that maternal obesity disrupts the normal development of these neuronal circuits [9,10,11]. However, the cellular mechanisms involved in hypothalamic development and how these mechanisms are perturbed in a context of maternal obesity remain elusive.

Endoplasmic reticulum (ER) stress provides an attractive mechanism in understanding the programming effects of maternal obesity on the bodily functions. Alterations in cellular homeostasis can lead to ER stress and the activation of the unfolded protein response (UPR) pathway. Previous studies have demonstrated that ER stress and UPR signaling pathway activation play important roles in obesity-induced insulin resistance and type 2 diabetes during adult life. Obesity caused by leptin deficiency or high-fat feeding in mice induces ER stress in peripheral tissues as well as in the hypothalamus [12,13,14]. Furthermore, relieving ER stress with chemical chaperones, i.e., agents that have the ability to increase ER folding machinery, increases insulin sensitivity and reverses type 2 diabetes in adult *ob/ob* mice while showing improved leptin sensitivity in adult obese mice fed a high-fat diet (HFD) [13,14]. Moreover, genetic manipulation of the UPR transcription factor spliced X-box binding protein (Xbp1) specifically in POMC neurons protects against diet-induced obesity and ameliorates leptin and insulin sensitivity [15]. Despite accumulating evidence supporting the role of ER stress in metabolic regulation, an association between maternal obesity, ER stress, and the programming of obesity and hypothalamic development has not yet been established.

In the present study, we investigated whether maternal diet-induced obesity induces ER stress during neonatal life in the offspring and how it contributes to the nutritional programming of obesity and hypothalamic development. We found that maternal obesity causes metabolic and neurodevelopmental alterations in the offspring, accompanied with elevated ER stress in the hypothalamus and pancreas during postnatal development. Moreover, we found that pharmacological inhibition of ER stress has long-term beneficial effects on body weight, body composition, energy balance, glucose homeostasis, leptin sensitivity, and POMC axonal projections in the offspring born to obese dams. Finally, our study reveals that the neurodevelopmental effects of maternal obesity likely involve direct inhibitory action of saturated fatty acids on arcuate axon growth.

## Results

### Maternal obesity causes metabolic disturbances in the offspring

**Fig 1:**
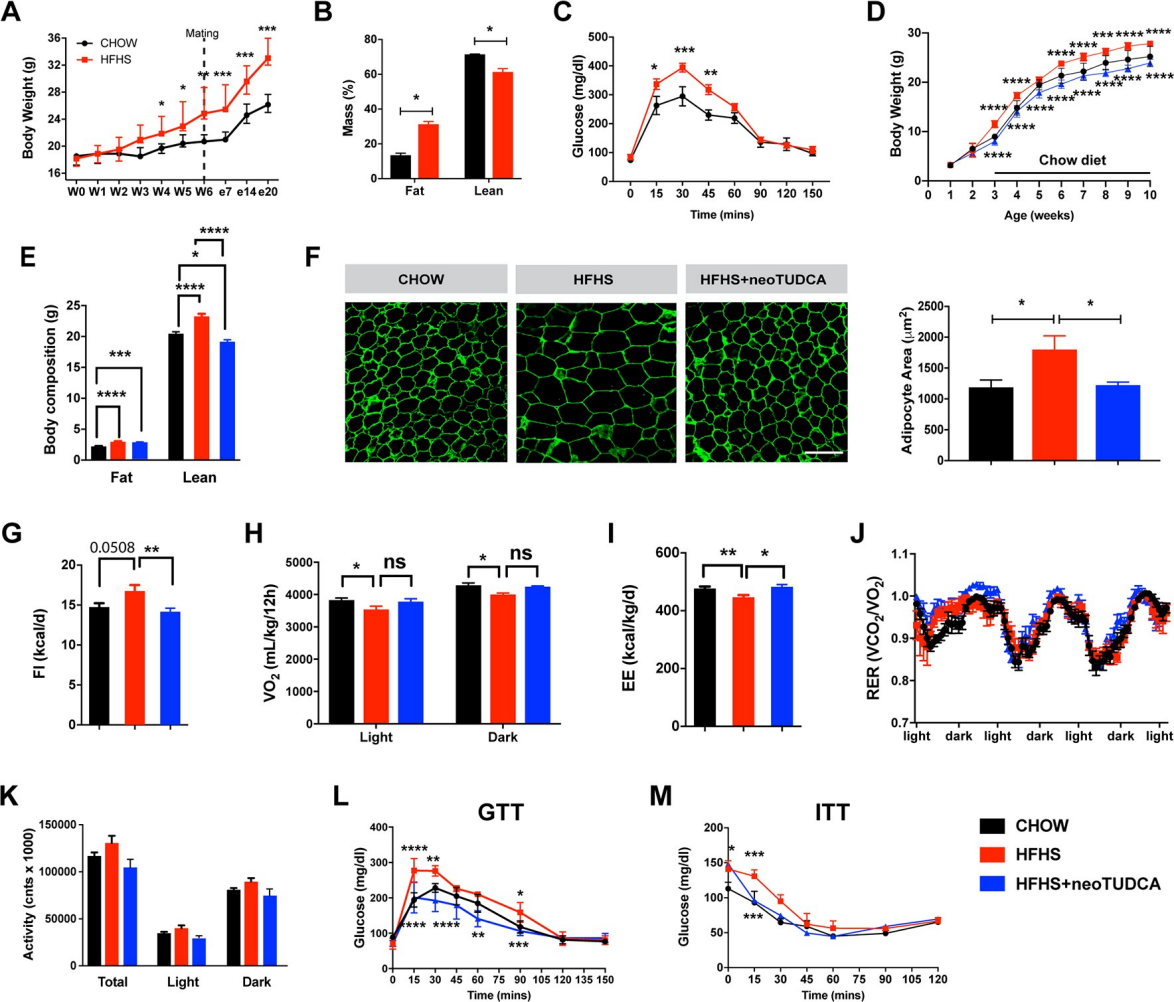
Maternal obesity impairs energy balance and glucose homeostasis in the offspring and neonatal TUDCA treatment improves this metabolic malprogramming. (A) Body weight curves of adult female mice fed a chow or an HFHS diet before and during pregnancy (*n =* 5 per group). (B) Body composition (*n =* 3 per group) and (C) GTT (*n =* 4–5 per group) of pregnant female mice fed a chow or HFHS diet at gestational day 16. (D) Body weight curves of mice born to chow-fed dams, HFHS-fed dams, or HFHS-fed dams and treated with neoTUDCA (*n =* 5–10 per group). (E) Average body composition (*n =* 5–8 per group) and (F) representative images and quantification of adipocyte size (immunostained for perilipin, green fluorescence) of 10-week-old mice born to chow-fed dams, HFHS-fed dams, or HFHS-fed dams and treated with TUDCA neonatally (*n* = 4–5 per group). (G) Food intake, (H) oxygen consumption, (I) energy expenditure, (J) RER, and (K) locomotor activity of 10-week-old mice born to chow-fed dams, HFHS-fed dams, or HFHS-fed dams and treated with TUDCA neonatally (*n =* 3–8 per group). (L) GTT and (M) ITT of 7- to 8-week-old mice born to chow-fed dams, HFHS-fed dams, or HFHS-fed dams and treated with TUDCA neonatally (*n =* 4–8 per group). Data are presented as mean ± SEM (panels A, C, D, J, L, M) or mean + SEM (panels B, E–I, K). *P ≤ 0.05, **P ≤ 0.01, ***P ≤ 0.001, and ****P ≤ 0.0001 versus chow groups. Statistical significance between groups was determined by one-way ANOVA (panels E–G, I–K), and two-way ANOVA (A–D, H, L, M) followed by Tukey’s Multiple Comparison test. Scale bar, 100 μm. The underlying data are provided in S1 Data. GTT, glucose tolerance test; HFHS, high-fat high-sucrose; ITT, insulin tolerance test; neoTUDCA, tauroursodeoxycholic acid given neonatally; RER, respiratory exchange ratio; TUDCA, tauroursodeoxycholic acid.

A mouse model of maternal obesity induced by high-fat high-sucrose (HFHS) feeding during pregnancy and lactation was used to study the effects of maternal obesity on the offspring’s metabolism and development. Adult female mice were either fed a HFHS (58% kcal fat with sucrose) or a control (chow) diet (6% calories from fat) 6 weeks before breeding. Dams were kept on their respective diet throughout pregnancy and lactation. A significant increase in dams’ weight gain was observed as early as 4 weeks after the HFHS diet began and persisted throughout the HFHS exposure (Fig 1A). This elevated body weight was associated with increased fat mass (Fig 1B). Moreover, dams fed a HFHS diet displayed altered glucose tolerance during gestation (Fig 1C).

Offspring born to these dams were fed a control diet after weaning. Male offspring born to the HFHS-fed dams had heavier body weights at weaning, with this elevated body weight persisting into adulthood (Fig 1D). In contrast, the female offspring born to the HFHS-fed dams showed no increase in body weight (S1 Fig). Therefore the remainder of the study was focused on the male offspring. Body composition evaluation showed that adult male mice born to obese dams displayed elevated fat and lean mass compared to adult mice born to lean dams (Fig 1E). Moreover, neonatal exposure to a HFHS diet caused adipocyte hypertrophy as revealed by a 1.5-fold increase in adipocyte size in epididymal white adipose tissue (Fig 1F). There was an increase in food intake and decrease in oxygen consumption (VO_2_) and energy expenditure in adult animals born to obese dams (Fig 1G–1I). Respiratory exchange ratio and locomotor activity were not significantly different compared to controls (Fig 1J and 1K). However, adult mice born to obese dams displayed impaired glucose and insulin tolerance compared to mice born to lean dams (Fig 1L and 1M).

### Maternal obesity induces ER stress during postnatal development and neonatal TUDCA treatment has long-term beneficial metabolic effects

**Fig 2:**
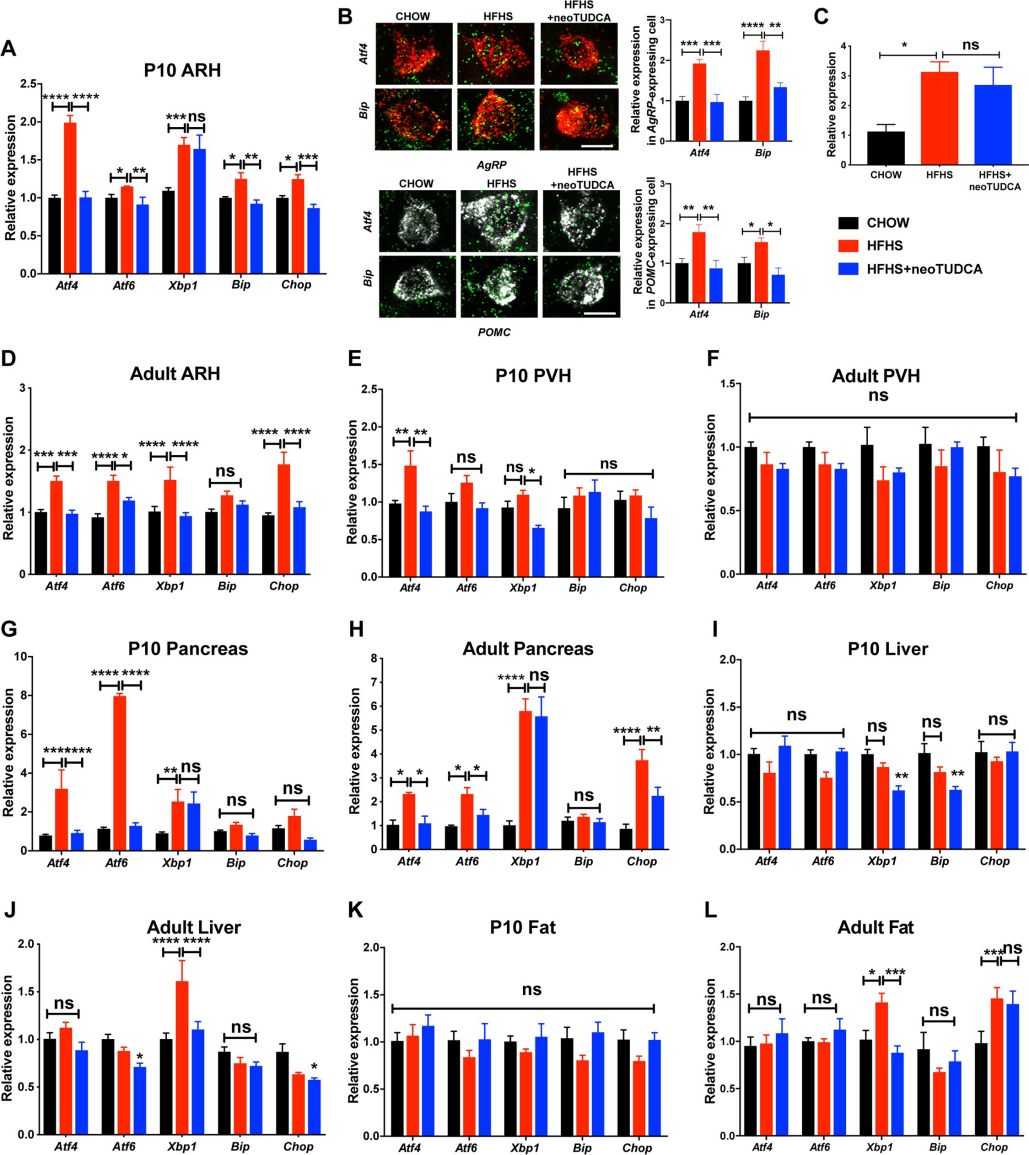
Neonatal TUDCA treatment reverses the elevated expression of ER stress markers in the offspring of obese dams. Relative expression of *Atf4*, *Atf6*, *Xbp1*, *Bip*, and *Chop* mRNA in panels A and D the ARH, (E, F) PVH, (G, H) pancreas, (I, J) liver, and (K, L) fat depot of (A, E, G, I, and K) P10 and (D, F, H, J, and L) adult 10-week-old mice born to chow-fed dams, HFHS-fed dams, or HFHS-fed dams and treated with TUDCA neonatally (*n =* 4–6 per group). (B) Representative images and quantification of *Atf4 and Bip* mRNA in arcuate *Pomc*- and *Agrp* mRNA-expressing cells of P10 mice born to chow-fed dams, HFHS-fed dams, or HFHS-fed dams and treated with TUDCA neonatally (*n* = 4–9 per group). (C) Spliced form of *Xbp1* in the ARH of P10 and mice born to chow-fed dams, HFHS-fed dams, or HFHS-fed dams and treated with TUDCA neonatally (*n* = 4–6 per group). Data are presented as mean + SEM. *P ≤ 0.05, **P < 0.01, ***P ≤ 0.001, and ****P ≤ 0.0001 versus other groups. Statistical significance between groups was determined by two-way ANOVA (panels A, B, D–L), and one-way ANOVA (panel C) followed by Tukey’s Multiple Comparison test. Scale bar, 5 μm. The underlying data are provided in S1 Data. ARH, arcuate nucleus; ER, endoplasmic reticulum; neoTUDCA, tauroursodeoxycholic acid given neonatally; PVH, paraventricular nucleus; TUDCA, tauroursodeoxycholic acid.

To examine if maternal obesity was associated with the activation of ER stress responses in the offspring, we measured the expression levels of the following ER stress markers in metabolically relevant tissues: activating transcription factor 4 (*Atf4)*, 6 (*Atf6)*, X-box binding protein (*Xbp1)*, glucose regulated protein GRP78 (referred to as *Bip*), and CCAAT-enhancer-binding protein homologous protein (*Chop*), in P10 and adult animals born to chow- or HFHS-fed dams. The mRNA levels of *Atf4*, *Atf6*, *Xbp1* (including the spliced form of *Xbp1*), *Bip*, and *Chop* were significantly elevated in the ARH of P10 mice born to obese dams (Fig 2A and 2C). We also assessed *Atf4* and *Bip* expression specifically in arcuate *Pomc* and *Agrp* neurons and found that the levels of these 2 ER stress markers were higher in these 2 neuronal populations in P10 mice born to obese dams (Fig 2B). Expression of *Atf4*, *Atf6*, *Xbp1*, and *Chop* mRNAs were significantly higher in the ARH of adult mice born to HFHS-fed dams (Fig 2D). In contrast, only *Atf4* mRNA levels were significantly increased in the paraventricular nucleus (PVH) of P10 mice (Fig 2E and 2F). *Atf4*, *Atf6*, and *Xbp1* mRNA levels were elevated in the pancreas of P10 pups of HFHS-fed dams (Fig 2G), but these markers were not significantly changed in neonatal liver and fat tissues (Fig 2I and 2K). In addition, *Atf4*, *Atf6*, *Xbp1*, *and Chop* mRNA levels were higher in the pancreas of adult mice born to obese dams (Fig 2H), but only *Xbp1* and *Xbp1* as well as *Chop* mRNA levels were significantly elevated in the liver and fat tissues, respectively, of adult mice born to HFHS-fed dams (Fig 2J and 2L).

To investigate the importance of early life ER stress, we treated pups born to HFHS-fed dams with daily peripheral injections of tauroursodeoxycholic acid (TUDCA) from P4 to P16, a period that is critical for growth and development, including that of the hypothalamus [2]. TUDCA is a chemical chaperone of low molecular weight that is known to alleviate ER stress [14,16]. Neonatal treatment with TUDCA in animals born to obese dams reversed induction of most ER stress markers in the postnatal and adult ARH, pancreas, liver, and adipose tissue (Fig 2A–2L), with the exception of *Xbp1* (including the spliced form of *Xbp1*) in the ARH and pancreas of P10 pups (Fig 2A, 2C and 2G) and in the pancreas of adult mice (Fig 2H), and *Chop* in adult adipose tissue (Fig 2L). Neonatal TUDCA treatment also reduced normal mRNA levels of *Xbp1* in the PVH and liver of P10 pups (Fig 2E and 2I), *Bip* in the liver of P10 pups (Fig 2I), and *Atf6* in the liver of adults (Fig 2J). Physiologically, neonatal TUDCA treatment in animals born to obese dams reversed alterations in body weight, body composition, adipocytes, food intake, and energy expenditure, as well as glucose and insulin tolerance (Fig 1D–1G, 1I and 1L–1M), with only VO_2_ showing no improvement (Fig 1H).

Together, these data indicate that maternal obesity causes elevated ER stress levels in metabolically relevant tissues during postnatal and adult life and that this induction of ER stress is reversible upon neonatal TUDCA treatment, which causes long-term beneficial effects on energy metabolism.

### Maternal obesity causes hyperleptinemia and reduces hypothalamic leptin sensitivity in the offspring that is reversible with neonatal TUDCA treatment

**Fig 3:**
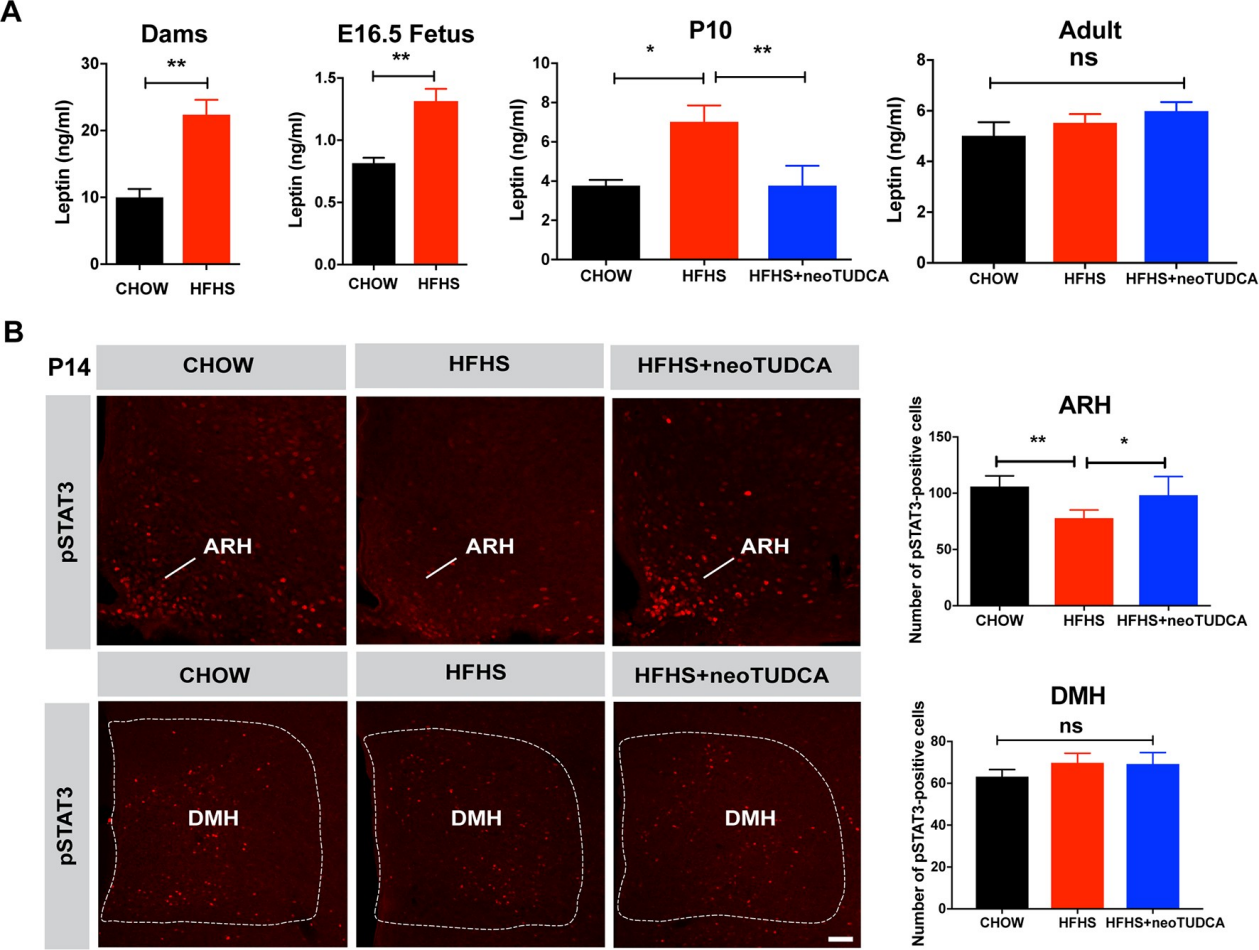
Maternal obesity causes neonatal hyperleptinemia and attenuated response to leptin that can be reversed by neonatal TUDCA treatment. (A) Serum leptin levels in dams at gestational day 16 and E16.5 fetuses of dams fed a chow or HFHS diet and in P10 and 10-week-old mice born to chow-fed dams, HFHS-fed dams, or HFHS-fed dams and treated with TUDCA neonatally (*n* = 4–8 per group). (B) Confocal images and quantification of the number of leptin-induced pSTAT3-immunoreactive cells in the ARH and DMH of P14 pups born to chow-fed dams, HFHS-fed dams, or HFHS-fed dams and treated with TUDCA neonatally (*n* = 5 per group). Data are presented as mean + SEM. *P ≤ 0.05 and **P < 0.01 versus chow groups. Statistical significance was determined by unpaired two-tailed Student t test (A), and one-way ANOVA followed by Tukey’s Multiple Comparison test (B). Scale bar, 100 μm. The underlying data are provided in S1 Data. ARH, arcuate nucleus; DMH, dorsomedial nucleus; HFHS, high-fat high-sucrose; neoTUDCA, tauroursodeoxycholic acid given neonatally; pSTAT3, phosphorylated signal transducer and activator of transcription 3; TUDCA, tauroursodeoxycholic acid.

Previous studies have reported that during perinatal life, leptin exerts marked neurodevelopmental and metabolic effects [8,17,18,19,20]. Therefore, we measured circulating leptin levels in animals exposed to maternal obesity. Maternal HFHS feeding was associated with a marked increase in serum leptin levels in dams at gestational day 16 and in E16.5 embryos (Fig 3A). Serum leptin levels were also elevated in P10 pups born to obese dams, which were normalized upon neonatal TUDCA treatment (Fig 3A). However, serum leptin levels were unchanged in adult mice born to HFHS-fed mothers (Fig 3A). Because leptin’s neurotrophic effects require intact ARH LepRb→pSTAT3 signaling [21], we also evaluated the number of pSTAT3-immunoreactive neurons after peripheral leptin injection and found that leptin treatment resulted in significantly fewer pSTAT3-positive cells in the ARH of P14 pups from obese dams and that neonatal TUDCA treatment enhanced ARH leptin-induced pSTAT3 (Fig 3B). To determine whether maternal obesity affected leptin sensitivity in other hypothalamic nuclei, we also examined leptin-induced pSTAT3-immunoreactivity in the dorsomedial nucleus of the hypothalamus (DMH) and found that the number of pSTAT3-positive cells was unaltered in the DMH of pups born to HFHS-fed dams (Fig 3B).

### Neonatal TUDCA exposure restores disrupted POMC axonal projections in the offspring of HFHS-fed dams

**Fig 4:**
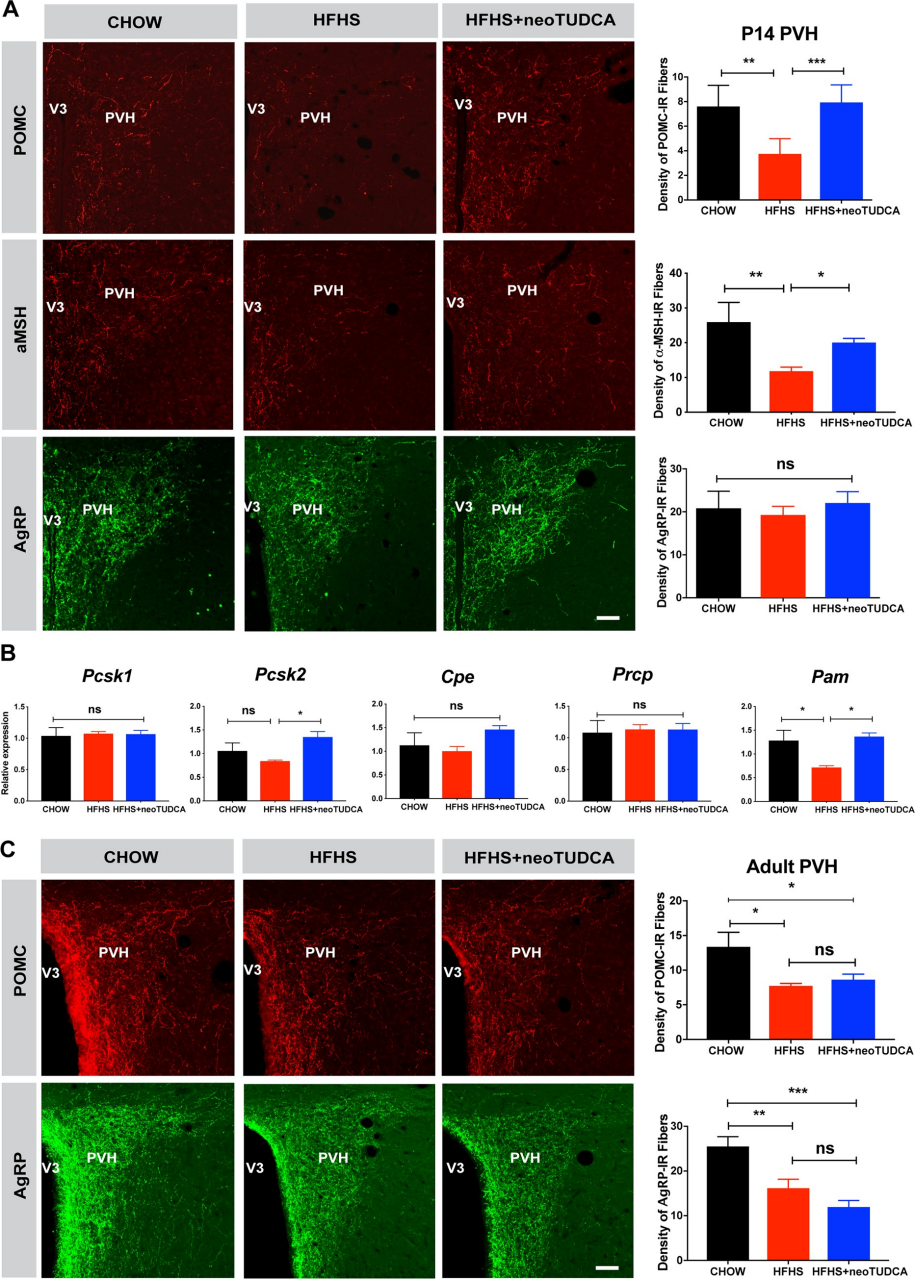
TUDCA treatment reverses neonatal disruption of POMC axonal projections induced by maternal obesity. (A) Confocal images and quantification of the density of POMC-, αMSH-, and AgRP-immunoreactive fibers in the PVH of P14 mice born to chow-fed dams, HFHS-fed dams, or HFHS-fed dams and treated with TUDCA neonatally (*n =* 5–7 per group). (B) Relative expression of *Pcsk1*, *Pcsk2*, *Cpe*, *Prcp*, and *Pam* in the ARH of P10 mice born to chow-fed dams, HFHS-fed dams, or HFHS-fed dams and treated with TUDCA neonatally (*n =* 6 per group). (C) Confocal images and quantification of the density of POMC-, and AgRP-immunoreactive fibers in the PVH of 10- to 12-week-old mice born to chow-fed dams, HFHS-fed dams, or HFHS-fed dams and treated with TUDCA neonatally (*n =* 5–7 per group). Data are presented as mean + SEM. *P ≤ 0.05, **P < 0.01, ***P ≤ 0.001, and ****P ≤ 0.0001 versus other groups. Statistical significance was determined by one-way ANOVA (A-B) followed by Tukey’s Multiple Comparison test. Scale bar, 50 μm. The underlying data are provided in S1 Data. AgRP, agouti-related peptide; ARH, arcuate nucleus of the hypothalamus; HFHS, high-fat high-sucrose; neoTUDCA, tauroursodeoxycholic acid given neonatally; POMC, pro-opiomelanocortin; PVH, paraventricular nucleus of the hypothalamus; TUDCA, tauroursodeoxycholic acid; αMSH, α-melanocyte-stimulating hormone.

During postnatal development, neuronal projections from the ARH reach their target nuclei, including the PVH, under the influence of leptin and leptin receptor signaling [8,21]. Because our results indicated that maternal obesity alters offspring’s leptin levels and ARH leptin signaling, we next investigated whether maternal obesity disrupts the development of ARH circuits by examining POMC/α-melanocyte-stimulating hormone (αMSH) and AgRP neuronal projections, two arcuate neuropeptidergic systems that play a critical role in energy balance. The density of POMC- and αMSH-immunoreactive fibers in the PVH of P14 mice born to obese dams was 1.5- and 2-fold lower than that observed in control mice (Fig 4A). In contrast, the density of AgRP-labeled projections innervating the PVH appeared normal in P14 pups born to HFHS-fed dams (Fig 4A). Also, the number *Pomc* and *Agrp* mRNA positive cells in the ARH of offspring born to obese mice was comparable to that of control mice (S2 Fig). The reduction in the density of POMC and αMSH fibers did not seem to be the consequence of defective expression of processing enzymes, because mRNA levels of proprotein convertase subtilisin/kexin type 1 (*Pcsk1*), proprotein convertase subtilisin/kexin type 2 (*Pcsk2*), carboxypeptidase E (*Cpe*), and prolylcarboxypeptidase (*Prcp*) were unchanged except for α-amidating monooxygenase (*Pam*) levels (an enzyme involed in in the maturation of αMSH) that were reduced in the ARH of P10 mice born to obese dams (Fig 4B). During adult life, both the densities of POMC- and AgRP-labeled fibers were also reduced (Fig 4C). Similar decreases in POMC and AgRP fiber densities were also observed in the adult DMH, which is another terminal field of ARH projections (S3 Fig).

Because TUDCA treatment restores normal leptin signaling in the developing ARH, we also examined whether neonatal TUDCA treatment improved ARH projections. Neonatal injections of TUDCA in pups born to obese dams restored a normal density of POMC- and αMSH-labeled fibers in P14 pups born to obese dams (Fig 4A). However, enhancing ER capacity neonatally did not influence POMC or AgRP projections in adult animals (Fig 4C and S3 Fig), which suggest that the neurodevelopmental effects of TUDCA are not permanent.

### Maternal obesity increases circulating fatty acids concentration and treatment with saturated fatty acids induces ER stress and blunts ARH axonal outgrowth

**Fig 5:**
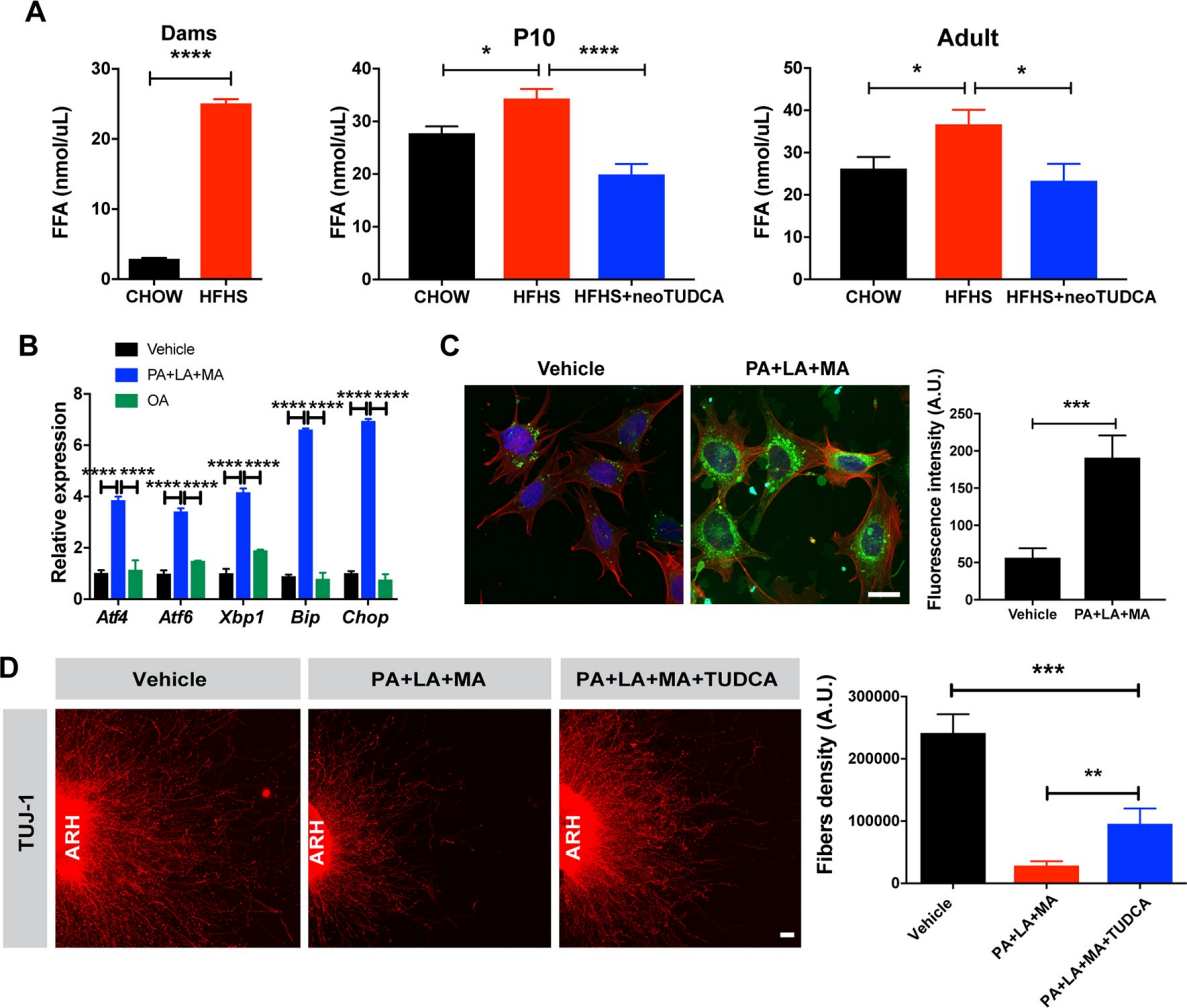
Saturated fatty acid treatment causes ER stress-induced disruption of axon growth. (A) Serum fatty acid levels in dams, P10 and 10-week-old mice born to chow-fed dams, HFHS-fed dams, or HFHS-fed dams and treated with TUDCA neonatally (*n =* 4–7 per group). (B) Relative expression of *Atf4*, *Atf6*, *Xbp1*, *Bip*, and *Chop* mRNA in mouse hypothalamic mHypoE-N43/5 cells treated with vehicle (BSA with 0.1% ethanol), or a cocktail of palmitate (250 μM) with lauric (1 mM) and myristic acids (200 μM) (PA+LA+MA), or OA alone for 24 h (*n =* 4–5 cultures per condition). (C) Representative images and quantification of the density of long-chain fatty acid analog BODIPY (green fluorescence) immunoreactivity in mHypoE-N43/5 cells treated with vehicle (BSA with 0.1% ethanol) or palmitate (250 μM) with lauric (1 mM) and myristic acids (200 μM) (PA+LA+MA) for 24 h (*n* = 5–7 cultures per condition). Red fluorescence and blue fluorescence depict actin filaments phalloidin and DAPI nuclear staining, respectively. (D) Confocal images and quantification of TUJ1 (neuron-specific class III beta-tubulin) immunoreactive fibers derived from isolated ARH explants incubated with vehicle (0.1% ethanol) or a combination of palmitate (250 μM) with lauric (1 mM) and myristic acids (200 μM) (PA+LA+MA) with or without TUDCA (750 μg/ml, *n* = 6 cultures per condition). Data are presented as mean + SEM. *P < 0.05, **P ≤ 0.01, ***P < 0.001 versus other groups. Statistical significance was determined by unpaired two-tailed Student t test (A, C, D), two-way ANOVA followed by Tukey’s Multiple Comparison test (B). Scale bars, 20 μm (C), and 50 μm (D). The underlying data are provided in S1 Data. ARH, arcuate nucleus; ER, endoplasmic reticulum; HFHS, high-fat high-sucrose; neoTUDCA, tauroursodeoxycholic acid given neonatally; OA, oleic acid; TUDCA, tauroursodeoxycholic acid; TUJ1, neuron-specific Class III β-tubulin.

Our results show that overconsumption of a HFHS diet during pregnancy and lactation is associated with abnormal hypothalamic development. Because this diet is rich in fatty acids (Table 1), we measured circulating fatty acid concentration during pregnancy and found that dams fed a HFHS diet had a 4-fold increase in serum fatty acid levels compared with dams fed a normal diet (Fig 5A). Offspring born to obese dams also displayed higher levels of circulating fatty acids at P10 that persisted into adulthood. Notably, neonatal TUDCA treatment restored normal levels of fatty acids (Fig 5A). To determine which type of fatty acids could cause neurodevelopmental abnormalities in our model, we reviewed the dietary fat content of the HFHS diet used in this study and found high concentrations (93.3%) of saturated fatty acids, including palmitic, lauric, and myristic acids and low concentrations (2.4%) of monounsaturated fats such as oleic acid (Table 1).

**Table 1:** Fatty acid composition of D12331 diet.

Fatty acid	gm/kg diet
C6, Caproic	2.0
C8, Caprylic	25.7
C10, Capric	19.7
C12, Lauric	158.7
C14, Myristic	60.0
C16, Palmitic	31.6
C18, Stearic	36.3
C18:1, Oleic	8.7
C18:2, Linoleic	13.5
C18:3, Linolenic	2.0

Direct exposure of mHypoE-N43/5 cells to individual saturated fatty acids such as palmitic, lauric, myristic acids, or a combination of these fatty acids increased ER stress marker gene expression (S4 Fig). In particular, the mRNA expression of *Atf4*, *Atf6*, *Xbp1*, *Bip*, and *Chop* increased from 4- to 7-fold in cells treated with a combination of palmitic, lauric, and myristic acids compared to vehicle-treated cells (Fig 5B). In contrast, expression of ER stress markers was not affected when cells were treated with the monousaturated fat oleic acid (Fig 5B).

We assessed fatty acids intracellular transport in hypothalamic cells using BODIPY, a fluorescent long-chain fatty acid analog. Exposure of hypothalamic mHypoE-N43/5 cells to a combination of palmitic, lauric, and myristic acids resulted in greater BODIPY labeling in mHypoE-N43/5 cell bodies compared to vehicle-treated cells (Fig 5C). In order to determine whether these saturated fatty acids also impacted ARH axon growth and affected ER stress, we also performed a series of in vitro experiments in which ARH explants were microdissected, placed in a collagen matrix, and then exposed to either a combination of saturated fatty acids (i.e., palmitic, lauric, and myristic acids), saturated fatty acids with TUDCA, or vehicle alone. After 48 hours, the density of TUJ1-labeled neurite, neuron-specific class III beta-tubulin, from ARH explants treated with saturated fatty acids was approximately 10-fold lower than that of vehicle-treated explants (Fig 5D). Moreover, pre-incubation of ARH explants with TUDCA improved disrupted axon outgrowth after saturated fatty acid treatment (Fig 5D).

Together, these data indicate that maternal obesity causes elevated circulating fatty acid levels in both the obese dams and offspring born to obese dams and that direct exposure to saturated fatty acids induced ER stress gene expression in hypothalamic cells. They also show that saturated fatty acids can be transported in hypothalamic cells, blunting axon growth, with this phenomenon appearing to involve ER stress pathways.

## Discussion

Although the link between perinatal overnutrition and lifelong metabolic regulation has been clearly shown, little is known about the mechanisms underlying this programming effect. In this study, we show that maternal obesity causes lifelong metabolic alterations associated with the abnormal development of hypothalamic feeding circuits in the offspring. We also report that maternal obesity induces ER stress in key tissues involved in energy metabolism during critical periods of growth and development, particularly in the arcuate nucleus and pancreas. Moreover, we found that relieving ER stress neonatally ameliorates metabolic and hypothalamic structural abnormalities in animals born to obese dams and that these effects are likely mediated through increased leptin sensitivity. Furthermore, we report that saturated fatty acids exert a direct inhibitory action on arcuate axon growth.

Our findings are generally consistent with previous studies demonstrating that maternal obesity causes lifelong weight gain and glucose intolerance associated with disruption in AgRP/NPY and POMC axonal projections during adulthood [10,11]. However, our study reveals that maternal obesity does not affect AgRP circuits during early postnatal life whereas it affects POMC axonal projections, suggesting that distinct mechanisms underlie the effects of maternal HFHS feeding on POMC neurons versus AgRP/NPY neurons. Vogt and colleagues have specifically attempted to compare the consequences of maternal obesity during gestation and lactation and have shown that maternal consumption of HFD during lactation (but not during pregnancy) is sufficient to cause obesity and diabetes, leading to alterations of the development of POMC projections in the offspring [10]. Consistent with the importance of the postnatal period in the nutritional programming of metabolism and hypothalamic circuits, exposure to chronic postnatal overnutrition by rearing neonates in small litters also predisposes them to obesity and disrupts hypothalamic development [22,23,24].

Our studies support the idea that maternal obesity alters growth of ARH axons to their target nuclei. Consistent with this hypothesis, the reduction in the density of POMC and αMSH-immunoreactive fibers is observed as early as P14, i.e., when arcuate neurons extend their axonal projections to their target nuclei [8]. Moreover, we found that the ability of ARH neurons to extend TUJ1^+^ fibers (a marker of axons independent of peptide content axons) is attenuated in explants derived exposed to saturated fatty acid. These findings suggest that the reduction in POMC^+^ fibers found in mutant mice is caused by a reduction in axon density as opposed to changes in the peptide content in axons. Consistent with this hypothesis, we found that expression of enzymes involved in POMC processing, such as *Pcsk1* and *Pcsk2*, was normal in animals born to HFHS dams.

A variety of developmental pathways control the development of arcuate feeding circuits. Among this array of signals, attention has been given to leptin. The density of ARH axonal projections is reduced in leptin-deficient mouse neonates and adults, which can be rescued with leptin treatment during early postnatal life [8,25,26]. Moreover, leptin appears to exert its neurodevelopmental actions on arcuate circuits though LepRb→pSTAT3 signaling [21]. The data presented here indicate that maternal obesity causes chronic hyperleptinemia in the offspring associated with reduced arcuate leptin-induced pSTAT3 during a critical period of hypothalamic development. A similar increase in circulating leptin levels and a reduction in arcuate leptin sensitivity has been reported in rat neonates exposed to chronic postnatal overnutrition [22]. However, the mechanisms underlying this early leptin resistance remain unclear. Here, we show that relieving ER stress enhances arcuate leptin resistance and improves hypothalamic development, leading to long-term metabolic outcomes. These findings are consistent with previous data showing that ER stress inducers, such as tunicamycin, blunts neurite elongation, and induce a collapse of neuronal growth cones from PC-12 cells or dissociated rat sensory neurons [27]. Remarkably, although TUDCA treatment restores normal POMC projections in neonates, the density of POMC fibers remain attenuated in adult mice treated with TUDCA neonatally. These results suggest that the effects of TUDCA are not permanent and that a longer TUDCA treatment might be required to cause a more sustain effect on POMC projections. These data also suggest that hypothalamic peptidergic pathways continue to remodel and change not just early in development but also during the postweaning period.

A limitation of the present study is that our animal model and experimental paradigm do not allow us to definitively distinguish the central versus peripheral sites of action of maternal HFHS diet and neonatal TUDCA treatment. However, TUDCA is a bile acid compound that has been shown to be permeable to the blood–brain barrier [28]. Therefore, although the exact site(s) of action of TUCDA remains to be determined, it might at least involve a direct effect on ARH neurons. Consistent with this idea, we report that TUDCA injections reversed increase in *Atf4* and *Bip* expression specifically in *Pomc* and *Agrp* neurons. Moreover, previous studies have reported that the pharmacological induction and genetic loss of the ER stress function in the brain block hypothalamic leptin-induced STAT3 activation [14].

Future studies are needed to determine the contribution of specific ER stress pathways to the nutritional programming of hypothalamic development and leptin resistance using, e.g., genetic approaches. However, previous studies have shown that overexpression of *Xbp1* or *Atf6* in mouse embryonic fibroblast cells increases their resistance to the inhibitory effects of tunicamycin and prevents ER stress‐mediated inhibition of leptin signaling [14]. Moreover, when fed an HFD, mice lacking *Xbp1* in neurons displayed an obesogenic phenotype, associated with hyperphagia and reduced oxygen consumption [14]. In addition, leptin‐induced STAT3 phosphorylation was significantly attenuated in the hypothalamus of these mice [14]. Furthermore, constitutive expression of a dominant *Xbp1* form specifically in POMC neurons lead to a lean phenotype, characterized by increased energy expenditure and leptin sensitivity, further supporting a fundamental role for the XBP1 pathway in POMC neurons in the deleterious metabolic effects of hypothalamic ER stress [15].

Our results show that lipid overload, especially saturated fatty acids, triggers ER stress in hypothalamic cells and that it contributes to disruption in arcuate axon growth. Previous studies have demonstrated that hypothalamic neurons can sense circulating fatty acids and that endogenous lipid metabolism in the hypothalamus is a key mechanism in regulating whole‐body energy balance [29]. Adult rodents fed an HFD exhibited elevated concentrations of fatty acids in the hypothalamus, which caused an accumulation of palmitoyl‐CoA and other harmful species [30,31]. Studies in hypothalamic cell lines have demonstrated that palmitate triggers ER stress and apoptosis [32,33,34]. In addition, intracerebroventricular injection of saturated fatty acids in vivo induces ER stress in the hypothalamus of rats [35]. Notably, palmitate decreases protein abundance and function of the α‐MSH receptor MC4-R while chemical chaperones reverse this biochemical abnormality [36], suggesting that saturated fatty acids may not only cause disruption in the development of POMC axonal projections but also attenuate the postsynaptic action of POMC-derived peptides through an ER stress-dependent mechanism.

## Methods and materials

### Ethics statement

All animal procedures were conducted in compliance with and approved by the Institutional Animal Care and Use Committee of the Saban Research Institute of the Children’s Hospital of Los Angeles. All experiments were performed in accordance with the relevant guidelines and regulations described in the IACUC-approved protocol number 230–19.

### Animals

The animals were housed under specific pathogen-free conditions, maintained in a temperature-controlled room with a 12 hour light/dark cycle, and provided ad libitum access to water and standard laboratory chow (Special Diet Services). At 7 weeks of age, female C57BL/6J wild-type (WT) mice were placed on either a regular chow diet [4.5 kcal% fat, provided by PicoLab Rodent Diet 5053] or a HFHS diet [58 kcal% fat with sucrose, provided by Research Diet D12331] for 6 weeks before mating. The mice were kept on their respective diets throughout pregnancy and lactation. Male breeders were fed a normal chow diet. Offspring were fed a normal chow diet after weaning. Litter sizes were standardized to 6 pups 48 hours postdelivery and attempts were made to maintain an equal sex ratio.

### Neonatal TUDCA treatment

The mice were injected intraperitoneally daily with TUDCA (150 mg/kg/day, Millipore, cat# 580549) from P4 to P16. Controls received injections with an equivalent volume of vehicle (0.9% NaCl).

### Tissue collection

The ARH and PVH of P10 and 10-week-old mice were dissected under a stereomicroscope. Liver, pancreas, and epididymal white adipose tissues were collected from P10 and 10-week-old mice.

### Embryo blood collection

Uterine horns containing the embryos from pregnant dams were removed and transferred to 10-cm Petri dishes containing cold PBS buffer. The uterine horns were then opened, and embryos were transferred to a new dish containing cold PBS buffer. The embryo head was then cut with a sharp razor blade and placed in a separate dish without buffer. Blood was then collected using a micropipette.

### Cell culture and fatty acid treatment

The embryonic mouse hypothalamic cell line mHypoE-N43/5 (Cedarlane, cat# CLU127) was cultured in Dulbecco’s modified Eagle’s medium (Sigma, D5796) supplemented with 10% fetal bovine serum, 100 U/ml penicillin and 100 μg/ml streptomycin at 37°C in 5% CO_2_ and a humidified atmosphere. mHypoE-N43/5 cells were plated out at density of 6 × 10^5^ cells per well in a 6-wells plate. The following day, medium was changed to culture medium containing either vehicle (BSA with 0.1% ethanol; Sigma), or palmitic (PA; 250 μM; Sigma, cat# P9767), lauric (LA; 1mM; Sigma, cat# W261416), myristic (MA; 200 μM; Sigma, cat# M3128), or oleic acids (OA; 250 μM; Sigma, cat# O7501), or combination of these fatty acids for 24 h.

### RNA extraction and RT-qPCR analyses

Total RNA was isolated using the Arcturus PicoPure RNA Isolation Kit (for hypothalamic samples) (Life Technologies), the RNeasy Lipid Tissue Kit (for peripheral samples) (Qiagen), or PureLink RNA mini kit (for mHypoE-N43/5 cell samples). cDNA was generated with the High-Capacity cDNA Reverse Transcription Kit (Life Technologies). Quantitative real-time PCR was performed using TaqMan Fast Universal PCR Master Mix (Thermo Fisher, cat# 4352042) and the commercially available TaqMan gene expression primers: *Atf4* (Mm00515324_m1), *Atf6* (Mm01295317_m1), *Xbp1* (Mm00457357_m1), spliced form of *Xbp1* (Mm03464496_m1), *Bip* (Mm00517691_m1), *Chop* (Mm00492097_m1), *Cpe* (Mm00516341_m1), *Pcsk1* (Mm00479023_m1), *Pcsk2* (Mm00500981_ m1), *Prcp* (Mm00804502_m1), *Pam* (Mm01293044_m1), and *Gapdh* (Mm99999915_g1). mRNA expression was calculated using the 2^-ΔΔCt^ method after normalization to the expression of the *Gapdh* housekeeping gene. All assays were performed using an Applied Biosystems 7900 HT real-time PCR system.

### Physiological measures

Maternal body weight was recorded weekly until the end of pregnancy. Offspring (*n =* 5 per group) were weighed weekly 1 to 10 weeks of age using an analytical balance. Body composition analysis (fat/lean mass) was performed on pregnant females at gestational day 16 and on the offspring at 10 weeks of age using NMR (Echo MRI). Food intake, O_2_ and CO_2_ production, energy expenditure, RER (i.e., VCO_2_/O_2_), and locomotor activity (XY) were monitored at 10 weeks of age using a combined indirect calorimetry system (TSE Systems). The mice were acclimated in monitoring chambers for 2 days, and the data were collected for 3 days.

Glucose and insulin tolerance tests (GTT and ITT) were conducted in 7- to 8-week-old mice through i.p. injection of glucose (1.5 mg/g body weight) or insulin (2 U/kg body weight) after overnight fasting. Blood glucose levels were measured at 0, 15, 30, 45, 60, 90, 120, and 150 min postinjection, as previously described [36].

Serum leptin levels were assayed in chow-fed or HFHS-fed mothers at gestational day 16, and in the offspring of chow- or HFHS-fed dams at E16.5, P10, and 10 weeks of age using a commercially available leptin ELISA kit (Millipore, cat# EZML-82K). Serum free fatty acid levels were assayed in chow-fed or HFHS-fed mothers at gestational day 16 and in the offspring of chow- or HFHS-fed dams at P10 and 10 weeks of age using a commercially available FFA kit (Abcam, cat# ab65341).

### POMC, αMSH, and AgRP immunohistochemistry

Ten- to twelve-week-old mice were perfused transcardially with 4% paraformaldehyde. The brains were then frozen, sectioned at 30-μm thickness, and processed for immunofluorescence using standard procedures [8,37]. The primary antibodies used for IHC were as follows: rabbit anti-POMC (1:20,000, Phoenix Pharmaceuticals, cat# H029-30), sheep anti-αMSH antibody (1:40,000, Millipore, cat# AB5087), and rabbit anti-AgRP (1:1,000, Phoenix Pharmaceuticals, cat# H003-53). The primary antibodies were visualized with Alexa Fluor 488 donkey anti-rabbit IgG (1:200, Millipore, cat# A21206), Alexa Fluor 568 donkey anti-sheep IgG (1:200, Millipore, cat# A21099), or Alexa Fluor 568 donkey anti-rabbit IgG (1:200, Millipore, cat# A10042). The sections were counterstained using bis-benzamide (1:10,000, Invitrogen, cat# H3569) to visualize cell nuclei.

### Multiplex fluorescent in situ hybridization

P10 and P14 mice were perfused with 4% paraformaldehyde. The brains were then frozen, sectioned at 20-μm thickness, and processed for multiplex fluorescent in situ hybridization. Commercially available RNAscope Multiplex Fluorescent reagent kits and RNAscope probes (*Agrp* #400711, *Pomc* #314081, *Atf4* #405101, *Bip* #438831) were used for transcript detection (Advanced Cell Diagnostics).

### pSTAT3 immunohistochemistry

Leptin (3 mg/kg; Peprotech, cat# 450–31) was injected intraperitoneally in P14 pups. Animals were perfused 45 minutes later with a solution of 2% paraformaldehyde. Frozen coronal sections were cut at 30 μm thickness and pretreated for 20 min with 0.5% NaOH and 0.5%H_2_O_2_ in KPBS, followed by immersion in 0.3% glycine for 10 min. Sections were then placed in 0.03% SDS for 10 minutes and placed in 4% normal serum + 0.4% Triton X-100 + 1% BSA (fraction V) for 20 minutes before incubation for 48 hours with a rabbit anti-pSTAT3 antibody (1:1,000, Cell Signaling, cat# 9131). The primary antibody was localized with Alexa Fluor 568 Goat anti-Rabbit IgGs (Invitrogen; 1:200, cat# A11036). Sections were counterstained using bis-benzamide (Invitrogen; 1:10,000, cat# H3569) to visualize cell nuclei and cover slipped with buffered glycerol (pH 8.5).

### Histomorphological assessment of white adipose tissue

Epididymal white adipose tissue from 10-week-old mice were collected, fixed in a 4% paraformaldehyde solution, sectioned at 5 μm thickness, and then stained with a Perilipin A/B antibody (1:1,000, Sigma, cat# P1873) using standard procedures.

### BODIPY staining

mHypoE-N43/5 cells were treated with vehicle or a combination of palmitic (250 μM; Sigma, cat# P9767), lauric (1mM; Sigma, cat# W261416), and myristic acids (200 μM; Sigma, cat# M3128) for 24 hours and 2 μM of Bodipy 493/503 (4,4-difluro-1,3,5,7-tetramethyl-4-bora-3a,4a-diaza-s-indacene; Invitrogen, cat# D3922) and Alexa Fluor 568 Phalloidin (0.1 μM; Invitrogen, cat# A12380) were added to culture media for 15 minutes at room temperature. mHypoE-N43/5 were then fixed in a solution of 4% paraformaldehyde for 5 minutes and washed with KPBS. Slides were counterstained using bis-benzamide (Invitrogen; 1:10,000, cat# H3569) to visualize cell nuclei.

### Isolated ARH explant culture

Brains were collected from P4 mice and sectioned at 200-μm thickness with a vibroslicer as previously described [8,37,38]. The ARH was then carefully dissected out of each section under a stereomicroscope. Explants were cultured onto a rat tail collagen matrix (BD Bioscience) and each explant was pretreated for 6 hours with fresh modified Basal Medium Eagle (Invitrogen) containing TUDCA (750μg/ml) or vehicle followed by a combination of palmitic (250 μM; Sigma, cat# P9767), lauric (1mM; Sigma, cat# W261416), and myristic acids (200 μM; Sigma, cat# M3128) or vehicle alone (BSA with 0.1% ethanol). After 48 hours, the explants were fixed in paraformaldehyde and neurites extending from the explants were stained with a TUJ1 (βIII tubulin) antibody (rabbit, 1:5,000, Covance, cat# MMS-435P) as described previously [37,38].

### Image analysis

The images were acquired using a Zeiss LSM 710 confocal system equipped with a 20X objective through the ARH (for cell numbers), through the PVH and the DMH (for fibers density), through adipose tissue (for adipocyte size), and through mHypoE-N43/5 cell cultures (for BODIPY staining). The average number of cells and density of fibers were analyzed in 2 to 4 sections per culture. For the explant experiments, sections were acquired using a Zeiss LSM 710 confocal system equipped with a 10X objective. Slides were numerically coded to obscure the treatment group. The image analysis was performed using ImageJ analysis software (NIH) as previously described [21,37,38].

For the quantitative analysis of cell number, *Pomc*, *Agrp*, and pSTAT3-positive cells were manually counted. Only cells with corresponding bis-benzamide-stained nuclei were included in our counts.

Determination of mean adipocyte size (μm^2^) was measured semi-automatically using the FIJI distribution of Image J software (NIH, ImageJ1.47i). The average adipocyte size measured from 3 fields and 6 sections in each mouse was used for statistical comparisons used for statistical comparisons.

For the quantitative analysis of fiber density (for POMC, αMSH, AgRP, and TUJ1 fibers) and BODIPY fluorescence, each image plane was binarized to isolate labeled materials from the background and to compensate for differences in fluorescence intensity. The integrated intensity, which reflects the total number of pixels in the binarized image, was then calculated for each image as previously described [8,37]. This procedure was conducted for each image plane in the stack, and the values for all of the image planes in a stack were summed.

### Statistical analysis

All values are represented as the mean ± SEM. Statistical analyses were conducted using GraphPad Prism (version 5.0a). Data sets with only 2 independent groups were analyzed for statistical significance using unpaired two-tailed Student *t* test. Data sets with more than 2 groups were analyzed using one-way ANOVA followed by the Tukey’s Multiple Comparison test. For statistical analyses of body weight, GTT, ITT, and RER, we performed two-way ANOVAs followed by Tukey’s Multiple Comparison test. Statistically significant outliers were calculated using Grubb’s test for outliers. *P* ≤ 0.05 was considered statistically significant.

## Supporting information

S1 Fig Maternal obesity does not affect body weight in the female offspring. Body weight curves of female mice born to chow- or HFHS-fed dams (*n =* 5 per group). Data are presented as mean ± SEM. Statistical significance was determined by a two-way ANOVA followed by Tukey’s Multiple Comparison test. The underlying data are provided as a Source Data file.
(PDF)

S2 Fig Maternal obesity does not affect *Pomc* or *Agrp* cell numbers. Representative image and quantification of the number of *Pomc*- and *Agrp* mRNA-expressing cells in the ARH of P14 neonates born to dams fed a chow or a HFHS diet (*n =* 6–9 per group). Data are presented as mean + SEM. Statistical significance was determined by unpaired two-tailed Student *t* test. Scale bar, 50 μm. The underlying data are provided as a Source Data file. ARH, arcuate nucleus of the hypothalamus; HFHS, high-fat high-sucrose.
(PDF)

S3 Fig TUDCA treatment does not reverse maternal obesity-induced disruption of POMC and AgRP axonal projections to the dorsomedial nucleus. Confocal images and quantification of the density of POMC- and AgRP-immunoreactive fibers in the DMH of 10- to 12-week-old mice born to chow-fed dams, HFHS-fed dams, or HFHS-fed dams and treated with TUDCA neonatally (*n* = 5–7 per group). Data are presented as mean + SEM. *P ≤ 0.05 and **P < 0.01 versus other groups. Statistical significance was determined by one-way ANOVA followed by Tukey’s Multiple Comparison test. Scale bar, 50 μm. The underlying data are provided as a Source Data file. AgRP, agouti-related peptide; DMH, dorsomedial nucleus of the hypothalamus; HFHS, high-fat high-sucrose; neoTUDCA, tauroursodeoxycholic acid given neonatally; POMC, pro-opiomelanocortin; TUDCA, tauroursodeoxycholic acid.
(PDF)

S4 Fig Saturated fatty acids induce ER stress on hypothalamic cells. Relative expression of (A) *Atf4*, (B) *Atf6*, (C) *Xbp1*, (D) *Bip*, and (E) *Chop* mRNA in mouse hypothalamic mHypoE-N43/5 cells treated with either vehicle (BSA with 0.1% ethanol), PA (250 μM), LA (1 mM), MA (200 μM), OA (250 μM), or a combination of these fatty acids for 24 hours (*n* = 4 cultures per condition). Data are presented as mean + SEM. *P ≤ 0.05, **P ≤ 0.01, ***P ≤ 0.001, and ****P ≤ 0.0001 versus vehicle group. Statistical significance was determined by one-way ANOVA followed by Tukey’s Multiple Comparison test. The underlying data are provided as a Source Data file. ER, endoplasmic reticulum; LA, lauric acid; MA, myristic acid; OA, oleic acid; PA, palmitic acid.
(PDF)

S1 Data Original data for the graphs in Figs 1–5 and S1–S4 Figs. Each tab includes data for the noted panels in Figs 1–5 and S1–S4 Figs.
(XLSX)

## Abbreviations

AgRP: agouti-related peptide
ARH: arcuate nucleus of the hypothalamus
Atf4: activating transcription factor 4
Atf6: activating transcription factor 6
Chop: CCAAT-enhancer-binding protein homologous protein
DMH: dorsomedial nucleus of the hypothalamus
ER: endoplasmic reticulum
HFD: high-fat diet
HFHS: high-fat high-sucrose
LA: lauric acid
MA: myristic acid
NPY: neuropeptide Y
OA: oleic acid
PA: palmitic acid
POMC: pro-opiomelanocortin
pSTAT3: phosphorylated signal transducer and activator of transcription 3
PVH: paraventricular nucleus
TUDCA: tauroursodeoxycholic acid
TUJ1: neuron-specific Class III β-tubulin
UPR: unfolded protein response
Xbp1: X-box binding protein
αMSH: α-melanocyte-stimulating hormone
