# Supplementary material for: Maternal obesity-induced endoplasmic reticulum stress causes metabolic alterations and abnormal hypothalamic development in the offspring
Source: PLoS Biol. 2020 Mar 12;18(3):e3000296. doi: 10.1371/journal.pbio.3000296 (PMC7067374; doi:10.1371/journal.pbio.3000296)
Supplement: S1 Fig — Body weight curves of female mice born to chow- or HFHS-fed dams (n = 5 per group). Data are presented as mean ± SEM. Statistical significance was determined by a two-way ANOVA followed by Tukey’s Multiple Comparison test. The underlying data are provided as a Source Data file. (PDF) [file pbio.3000296.s001.pdf]

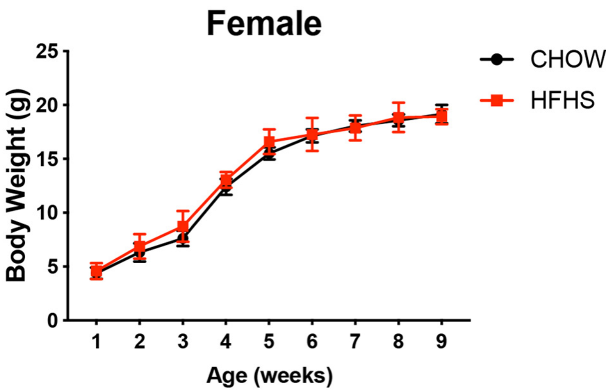

S1 Fig. Maternal obesity does not affect body weight in the female offspring. Body weight curves of female mice born to chow- or HFHS-fed dams (n = 5 per group). Data are presented as mean ± SEM. Statistical significance was determined by a two-way ANOVA followed by Tukey's Multiple Comparison test. The underlying data are provided in S1 Data.
