# Supplementary material for: Maternal obesity-induced endoplasmic reticulum stress causes metabolic alterations and abnormal hypothalamic development in the offspring
Source: PLoS Biol. 2020 Mar 12;18(3):e3000296. doi: 10.1371/journal.pbio.3000296 (PMC7067374; doi:10.1371/journal.pbio.3000296)
Supplement: S2 Fig — Representative image and quantification of the number of Pomc- and Agrp mRNA-expressing cells in the ARH of P14 neonates born to dams fed a chow or a HFHS diet (n = 6–9 per group). Data are presented as mean + SEM. Statistical significance was determined by unpaired two-tailed Student t test. Scale bar, 50 μm. The underlying data are provided as a Source Data file. ARH, arcuate nucleus of the hypothalamus; HFHS, high-fat high-sucrose. (PDF) [file pbio.3000296.s002.pdf]

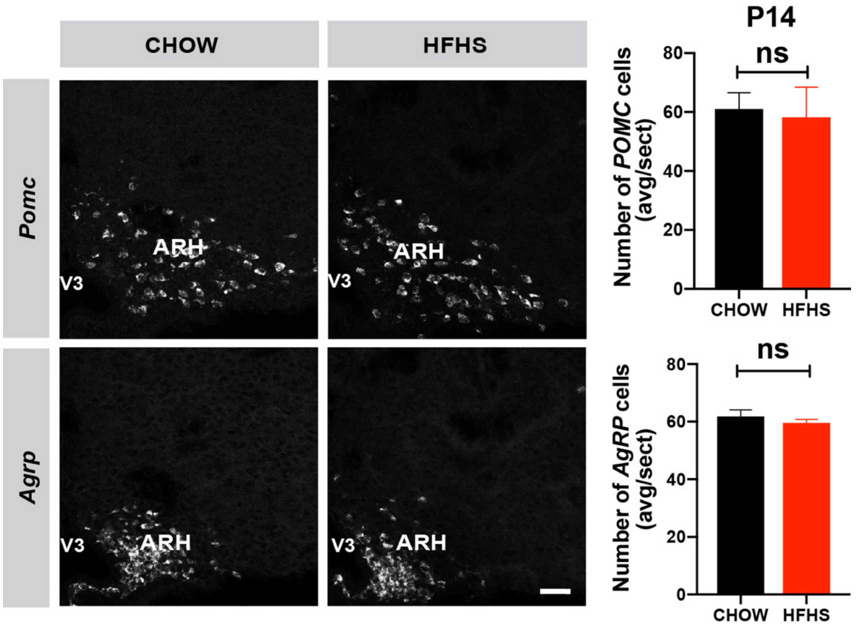

S2 Fig. Maternal obesity does not affect *Pomc* or *Agrp* cell numbers. Representative image and quantification of the number of *Pomc*- and *Agrp* mRNA-expressing cells in the ARH of P14 neonates born to dams fed a chow or a HFHS diet (n = 6-9 per group). Data are presented as mean + SEM. Statistical significance was determined by unpaired two-tailed Student's t test. Scale bar, 50 μm. The underlying data are provided in S1 Data.
