# Supplementary material for: Maternal obesity-induced endoplasmic reticulum stress causes metabolic alterations and abnormal hypothalamic development in the offspring
Source: PLoS Biol. 2020 Mar 12;18(3):e3000296. doi: 10.1371/journal.pbio.3000296 (PMC7067374; doi:10.1371/journal.pbio.3000296)
Supplement: S3 Fig — Confocal images and quantification of the density of POMC- and AgRP-immunoreactive fibers in the DMH of 10- to 12-week-old mice born to chow-fed dams, HFHS-fed dams, or HFHS-fed dams and treated with TUDCA neonatally (n = 5–7 per group). Data are presented as mean + SEM. *P ≤ 0.05 and **P < 0.01 versus other groups. Statistical significance was determined by one-way ANOVA followed by Tukey’s Multiple Comparison test. Scale bar, 50 μm. The underlying data are provided as a Source Data file. AgRP, agouti-related peptide; DMH, dorsomedial nucleus of the hypothalamus; HFHS, high-fat high-sucrose; neoTUDCA, tauroursodeoxycholic acid given neonatally; POMC, pro-opiomelanocortin; TUDCA, tauroursodeoxycholic acid. (PDF) [file pbio.3000296.s003.pdf]

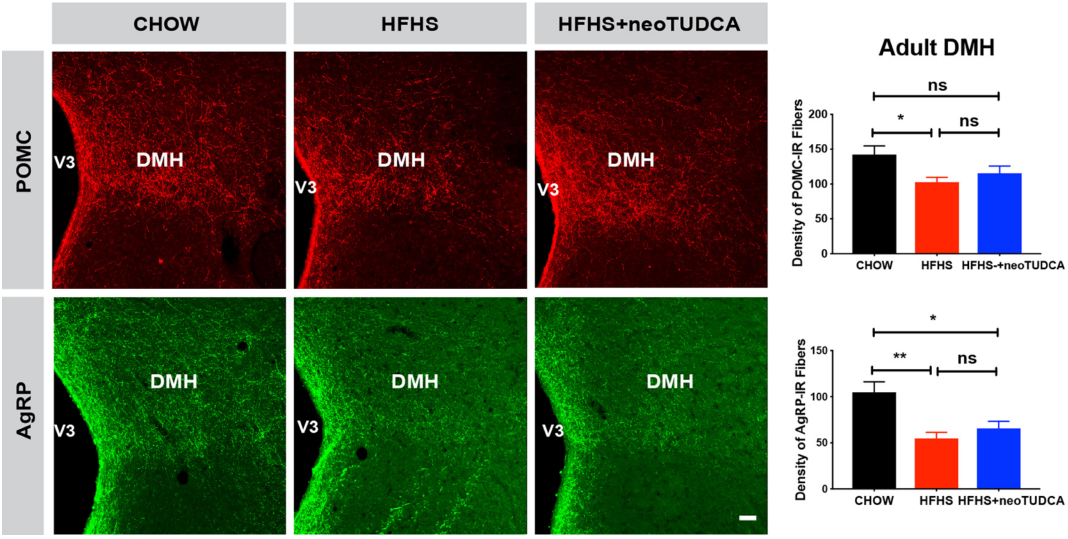

S3 Fig. TUDCA treatment does not reverse maternal obesity-induced disruption of POMC and AgRP axonal projections to the dorsomedial nucleus. Confocal images and quantification of the density of pro-opiomelanocortin (POMC)- and agouti-related peptide (AgRP)-immunoreactive fibers in the dorsomedial nucleus (DMH) of 10- to 12-week-old mice born to chow-fed dams, HFHS-fed dams, or HFHS-fed dams and treated with TUDCA neonatally (n = 5-7 per group). Data are presented as mean ± SEM. $^*P \leq 0.05$, and $^{**}P < 0.01$ *versus* other groups. Data are presented as mean + SEM. Statistical significance was determined by one-way ANOVA followed by Tukey's Multiple Comparison test. Scale bar, 50 μm. The underlying data are provided in S1 Data.
