# Supplementary material for: Maternal obesity-induced endoplasmic reticulum stress causes metabolic alterations and abnormal hypothalamic development in the offspring
Source: PLoS Biol. 2020 Mar 12;18(3):e3000296. doi: 10.1371/journal.pbio.3000296 (PMC7067374; doi:10.1371/journal.pbio.3000296)
Supplement: S4 Fig — Relative expression of (A) Atf4, (B) Atf6, (C) Xbp1, (D) Bip, and (E) Chop mRNA in mouse hypothalamic mHypoE-N43/5 cells treated with either vehicle (BSA with 0.1% ethanol), PA (250 μM), LA (1 mM), MA (200 μM), OA (250 μM), or a combination of these fatty acids for 24 hours (n = 4 cultures per condition). Data are presented as mean + SEM. *P ≤ 0.05, **P ≤ 0.01, ***P ≤ 0.001, and ****P ≤ 0.0001 versus vehicle group. Statistical significance was determined by one-way ANOVA followed by Tukey’s Multiple Comparison test. The underlying data are provided as a Source Data file. ER, endoplasmic reticulum; LA, lauric acid; MA, myristic acid; OA, oleic acid; PA, palmitic acid. (PDF) [file pbio.3000296.s004.pdf]

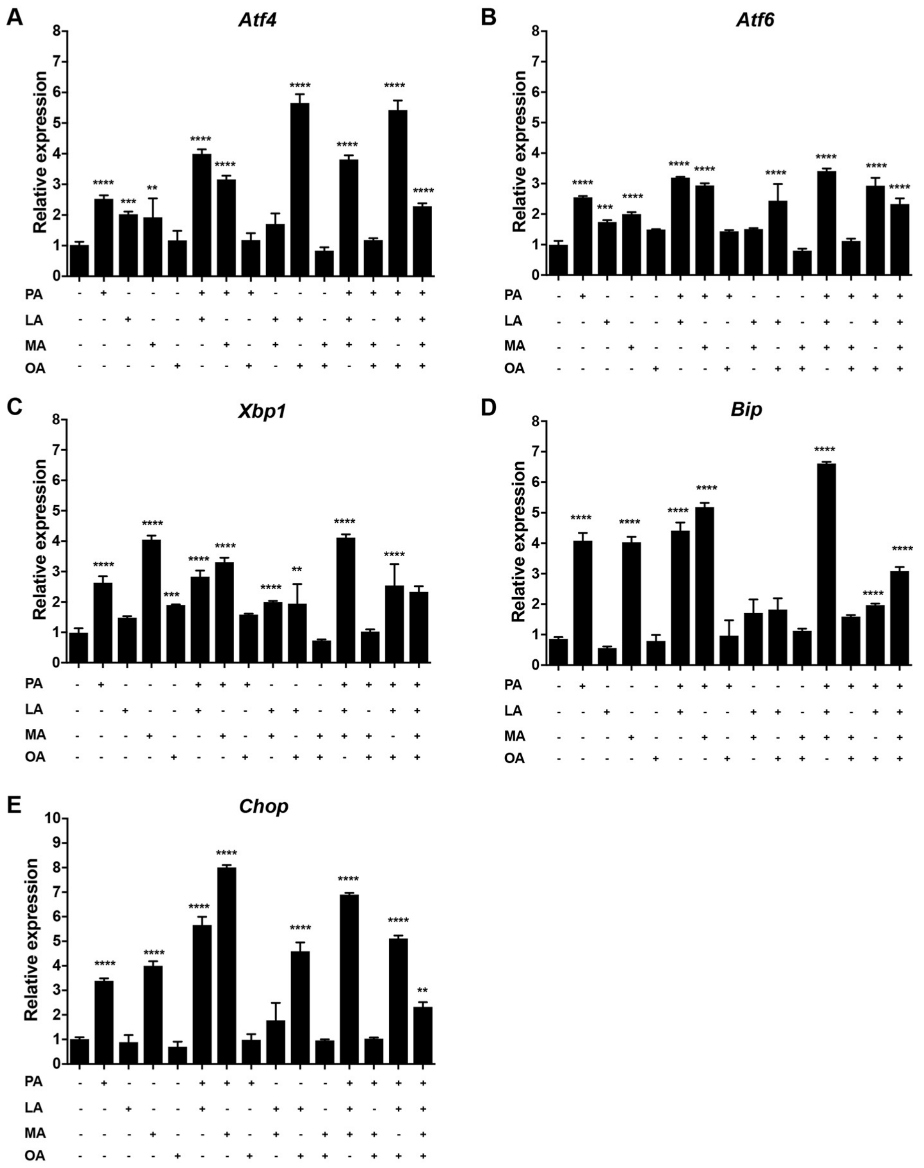

S4 Fig. Saturated fatty acids induce ER stress on hypothalamic cells. Relative expression of (A) *Atf4,* (B) *Atf6,* (C) *Xbp1,* (D) *Bip*, and (E) *Chop* mRNA in mouse hypothalamic mHypoE-N43/5 cells treated with either vehicle (BSA with 0.1% ethanol), palmitic (PA; 250 μM), lauric (LA; 1mM), myristic (MA; 200 μM), oleic acids (OA; 250 μM), or a combination of these fatty acids for 24 hours (n = 4 cultures per condition). Data are presented as mean + SEM. **P* ≤ 0.05, ***P* ≤ 0.01, ****P* ≤ 0.001, and *****P* ≤ 0.0001 *versus* vehicle group. Statistical significance was determined by one-way ANOVA followed by Tukey's Multiple Comparison test. The underlying data are provided in S1 Data.
